# Anaerobic Spondylodiscitis due to *Fusobacterium* Species: A Case Report Review of the Literature

**DOI:** 10.1155/2015/759539

**Published:** 2015-04-27

**Authors:** Tiffany N. Latta, Aimee L. Mandapat, Joseph P. Myers

**Affiliations:** ^1^Department of Medicine, Summa Akron City Hospital, Akron, OH 44304, USA; ^2^Division of Infectious Diseases, Department of Medicine, Summa Akron City Hospital, Akron, OH 44304, USA; ^3^Section of Infectious Diseases, Department of Internal Medicine, Northeast Ohio Medical University, Rootstown, OH 44272, USA

## Abstract

Spondylodiscitis caused by *Fusobacterium* species is rare. Most cases of spontaneous spondylodiscitis are caused by *Staphylococcus aureus* and most postoperative cases are caused by *Staphylococcus aureus* or coagulase-negative staphylococci. *Escherichia coli* is the most common Gram-negative organism causing spondylodiscitis. *Fusobacterium* species are unusual causes for anaerobic spondylodiscitis. We report the case of a patient with spontaneous L2-L3 spondylodiscitis, vertebral osteomyelitis, and epidural abscess caused by *Fusobacterium* species and review the literature for patients with *Fusobacterium* spondylodiscitis.

## 1. Introduction

Spondylodiscitis due to* Fusobacterium* species is unusual with only 15 cases having been previously reported [1–13]. Hematogenous spondylodiscitis is most often caused by* Staphylococcus aureus* [14–16]. Postoperative spondylodiscitis is most commonly due to staphylococcal species [17]. The most common etiologic agent of spontaneous Gram-negative spondylodiscitis is* Escherichia coli* [18].* Propionibacterium* and* Bacteroides* species are the most commonly reported causes for anaerobic spondylodiscitis [19]. We report a patient with spontaneous L2-L3 spondylodiscitis due to* Fusobacterium* species and review the literature for other reports of this disease.

## 2. Materials and Methods

PubMed and Google Scholar searches were performed using the words discitis, diskitis, spondylodiscitis, vertebral osteomyelitis, and* Fusobacterium* looking for previously reported cases of discitis and osteomyelitis caused by* Fusobacterium* species. References from each of the articles obtained by these searches were also reviewed for further pertinent case reports.

## 3. Case Report

A 57-year-old man with a past history of asthma was admitted to the hospital with a three-month history of gradually worsening low back pain aggravated by movement and not improved with rest, cyclobenzaprine, or a tapering course of prednisone. Physical examination revealed multiple abscessed teeth and tenderness to palpation over the second and third lumbar vertebrae. The neurologic examination was normal. Complete blood count revealed a WBC of 13,600/cmm (normal: 4.400–11,300/cmm) with 93.4% granulocytes, 5.6% lymphocytes, and 1.0% monocytes. Erythrocyte sedimentation rate was 6 mm/hr (normal: 0–10 mm/hr). C-reactive protein was 3.68 mg/dL (normal: <0.6 mg/dL). MRI of the lumbar spine revealed severe L2-L3 discitis, contiguous L2 and L3 vertebral osteomyelitis with end-plate erosion, and epidural and psoas muscle abscesses. CT-guided biopsy was performed and a pure culture of* Fusobacterium* species was isolated from anaerobic cultures of the disc-space tissue aspirate. Fungal and mycobacterial cultures were negative. The abscessed teeth were extracted and the patient was successfully treated with ertapenem, one gram intravenously daily for 8 weeks. Follow-up examination 6 months later showed mild residual pain and a normal physical examination.

## 4. Discussion

*Fusobacterium* is a genus of obligately anaerobic filamentous Gram-negative rods that are members of the phylum Fusobacteria [20, 21]. They can be divided into 13 species that inhabit the oral, gastrointestinal, upper respiratory tract, and vaginal mucosa [22].* Fusobacterium* species can be variable in Gram stain morphology and display a range of cellular morphologies from coccoid, pleomorphic spherules to rod shaped organisms. Rods can be short with rounded ends or long with pointed ends. Most* Fusobacterium* species are indole positive and produce butyric acid during the fermentation of glucose [20, 21].* Fusobacterium nucleatum* is the species most commonly described causing human infection and Lemierre's syndrome caused by* Fusobacterium necrophorum* is the best known infection associated with the Fusobacteria [23, 24]. Fusobacterial infections of almost every anatomic site have been reported [23, 24]. Bacteremia caused by* Fusobacterium* species is unusual but not rare [25–27]. Several patients with fusobacterial vertebral and paravertebral infections without classic spondylodiscitis have also been reported [28–31].

Patients with fusobacterial spondylodiscitis reported in the literature are listed in Table 1. Patient ages ranged from 8 to 84 years with a mean of 54.5 years. There were 12 men (75%) and 4 women (25%). The most common spinal level for spondylodiscitis was the lumbar area. There were 4 patients with thoracic disease (25%) and 12 with lumbar disease (75%). L3-L4 and L4-L5 discitis were most common with 4 patients with disease at each of those sites. One patient had multilevel disease (T6–T8). Three patients had no underlying illness. Seven of 16 patients (43.75%) had significant oral pathology: 5 with periodontitis and 2 with dental root abscess.* Fusobacterium* spondylodiscitis was microbiologically confirmed by blood culture alone in 6 cases (37.5%), by tissue biopsy culture in 8 cases (50.0%), and by blood and tissue biopsy cultures in 2 cases (12.5%). The duration of antimicrobial therapy was unknown in 2 patients and ranged from 6 to 19 weeks in the other 14 patients with an average of 11.1 weeks. Treatment regimens included penicillin, metronidazole, clindamycin, carbapenem, or some combination of these agents. Details of treatment are presented in Table 1. All 15 patients for whom information was available survived. Three patients required surgical intervention: one had drainage of an epidural abscess and anterior spinal stabilization; one had urgent laminectomy and drainage of an epidural abscess; and one had disc-space debridement and a spinal fusion.

*Fusobacterium* is an unusual cause of infectious spondylodiscitis but responds well to medical/surgical therapy with no deaths recorded in the reported patients. Blood, disc tissue, or psoas abscess cultures in our patients often required 72 hours of incubation before there was sufficient growth to identify a pathogen. Thus, any patient suspected of having culture-negative spondylodiscitis should have anaerobic cultures performed with an adequate time allowed for incubation of biopsy specimens before one proceeds with a detailed work-up for other etiologies of culture-negative disease. Treatment with at least 6 weeks of antimicrobial therapy was curative in all reported patients, although three patients required surgical intervention for complications such as paraspinal phlegmon.* Fusobacterium* isolates are usually sensitive to penicillin, ampicillin, amoxicillin/clavulanate, piperacillin/tazobactam, clindamycin, metronidazole, moxifloxacin, tigecycline, imipenem, and ertapenem [20, 21]. Because* Fusobacterium* spp. are often part of polymicrobial infectious flora, therapy should be guided by both available sensitivity testing and the potential presence of other aerobic/anaerobic organisms in cultures from the site of infection. Because of its once daily administration schedule and its antimicrobial spectrum, ertapenem is an especially attractive treatment option when outpatient parenteral antimicrobial therapy (OPAT) is being used. Moxifloxacin, a fluoroquinolone with high oral bioavailability, once daily administration, and aerobic/anaerobic spectrum, is an attractive oral alternative to OPAT with its requisite peripherally inserted central venous catheter [20, 21].

## Figures and Tables

**Table 1:** Spondylodiscitis caused by *Fusobacterium* species. Review of the literature.

Author [reference]	Year	Age (years)	Sex	Spinal level(s)	Underlying conditions	Microbiologic diagnosis	Antimicrobial therapy	Length of antimicrobial therapy	Surgical intervention	Outcome
Fain et al. [4]	1989	67	F	L1-L2	Cirrhosis; dental abscesses	Blood cultures: *F. nucleatum *	Amoxicillin-clavulanate PO + metronidazole PO × 6 weeks	6 weeks	None	Lived

Rubin et al. [11]	1991	58	M	T12-L1	Diabetes mellitus, periodontitis	Disc biopsy (puncture): *Fusobacterium* sp. + *S. aureus* + *S*. *sanguis* type II	Clindamycin (route of administration not specified) × 8 weeks	8 weeks	Dental extractions	Lived

Soubrier et al. [12]	1995	63	M	T10-T11	None	Blood cultures: *F. nucleatum *	Penicillin G IV + metronidazole IV × 3 days and **then** penicillin V PO × 2 months	8 weeks	None	Lived

Wang et al. [13]	1996	48	M	L1-L2	Periodontitis	Blood cultures: *F. nucleatum *	Penicillin G IV × 28 days and **then** clindamycin PO × 8 weeks	12 weeks	Dental extractions	Lived well 20 months later

Pampliega-Martinez et al. [9]	1997	84	M	T6-T7	Thoracic contusion	Disc aspirate: *F*. *necrophorum *	Amoxicillin PO × 46 days	8 weeks	None	Lived well 3 years later

de Gans et al. [3]	2000	53	F	L4-L5	Otitis media 5 months previously	Disc aspirate: *F. varium *	Amoxicillin-clavulanate PO × 8 weeks	8 weeks	Drainage of epidural abscess via anterior approach with anterior spinal stabilization	Lived well 6 months later

Abele-Horn et al. [1]	2001	20	M	L4-L5	*Mycoplasma pneumonia* pneumonia 3 weeks previously	Blood cultures: *F. necrophorum *	Ampicillin/sulbactam IV × 3 days and **then** imipenem-cilastatin IV × 4 weeks and **then** clindamycin PO × 3 months	19 weeks	None	Lived well 1 year later

Brook [2]	2001	8	M	L3-L4	Upper respiratory infection 27 days previously	Disc aspirate: *F. nucleatum *	Clindamycin IV × 3 weeks and **then** clindamycin PO × 3 weeks	6 weeks	None	Lived well 2 years later

Le Moal et al. [8]	2005	78	F	L5-S1	Periodontitis requiring right inferior molar extraction 3 months previously	Disc aspirate **and** blood cultures: *F. necrophorum *	Clindamycin IV × 4 weeks and **then** clindamycin PO × 8 wks.	12 weeks	None	Lived well 2 years later

Le Moal et al. [8]	2005	62	M	L4-L5	None	Blood cultures: *F. necrophorum *	Clindamycin IV × 4 weeks and **then** clindamycin PO × 8 weeks	12 weeks	None	Lived well 2 years later

Le Moal et al. [8]	2005	61	M	T6–T8	Diabetes mellitus; periodontal disease	Bone aspirate **and** blood cultures: *F*. *nucleatum *	Penicillin G IV × 4 weeks and **then** clindamycin PO × 8 weeks	12 weeks	Dental extractions	Lived well 3 years later

Goolamali et al. [5]	2006	63	M	L4-L5	Dental root abscess	Disc aspirate **and** open biopsy: *F*. *nucleatum *	Flucloxacillin IV + fusidic acid IV and **then** penicillin G IV + metronidazole (unknown route of administration)	Unknown	Right L5 laminectomy + drainage of multilevel epidural abscess	Lived well 2 months later

Joosten et al. [7]	2011	70	M	L3-L4	None	Blood cultures: *F. nucleatum *	Amoxicillin-clavulanate IV × 1 day, **then** ceftriaxone IV + metronidazole PO × 3 days, **then** metronidazole PO × 3.5 weeks, and **then** clindamycin PO × 14.5 weeks	19 weeks	None	Lived well 6 wks. after treatment

Ramos et al. [10]	2013	42	F	L3-L4	Crohn's disease	Disc aspirate: *F. nucleatum *	Ertapenem IV + metronidazole PO × 8 weeks and **then** amoxicillin-clavulanate PO × 10 weeks	18 weeks	None	Lived well 7 months later

Griffin and Christensen [6]	2014	38	M	L3-L4	Gingivitis; contusions after all-terrain vehicle rollover	Open biopsy: *F. nucleatum *	Ertapenem IV × 8 weeks and **then** amoxicillin PO indefinitely	Indefinite suppressive therapy	Debridement and spinal fusion of L2–L5	Unknown

Latta et al. [this case]	2015	57	M	L2-L3	Asthma	Disc aspirate: *Fusobacterium* species	Ertapenem IV × 8 weeks	8 weeks	None	Lived well 3 months after treatment
